# Using the Benford’s Law as a First Step to Assess the Quality of the Cancer Registry Data

**DOI:** 10.3389/fpubh.2016.00225

**Published:** 2016-10-13

**Authors:** Emanuele Crocetti, Giorgia Randi

**Affiliations:** ^1^Health in Society Unit, Directorate F Health, Consumers and Reference Materials, Joint Research Centre (JRC), European Commission, Ispra, Italy

**Keywords:** cancer registry, incidence, data quality, Benford, methodology

## Abstract

**Background:**

Benford’s law states that the distribution of the first digit different from 0 [first significant digit (FSD)] in many collections of numbers is not uniform. The aim of this study is to evaluate whether population-based cancer incidence rates follow Benford’s law, and if this can be used in their data quality check process.

**Methods:**

We sampled 43 population-based cancer registry populations (CRPs) from the Cancer Incidence in 5 Continents-volume X (CI5-X). The distribution of cancer incidence rate FSD was evaluated overall, by sex, and by CRP. Several statistics, including Pearson’s coefficient of correlation and distance measures, were applied to check the adherence to the Benford’s law.

**Results:**

In the whole dataset (146,590 incidence rates) and for each sex (70,722 male and 75,868 female incidence rates), the FSD distributions were Benford-like. The coefficient of correlation between observed and expected FSD distributions was extremely high (0.999), and the distance measures low. Considering single CRP (from 933 to 7,222 incidence rates), the results were in agreement with the Benford’s law, and only a few CRPs showed possible discrepancies from it.

**Conclusion:**

This study demonstrated for the first time that cancer incidence rates follow Benford’s law. This characteristic can be used as a new, simple, and objective tool in data quality evaluation. The analyzed data had been already checked for publication in CI5-X. Therefore, their quality was expected to be good. In fact, only for a few CRPs several statistics were consistent with possible violations.

## Introduction

The Benford’s law (1), originally identified by Newcomb (2), states that in many numerical series the distribution of the first significant digits (FSDs) (the first non-zero digit on the left side of a number) is not uniform. In fact, for numbers which adhere to this law, the probability of 1 to be the FSD is 30.1%, and this probability steadily decreases for the following digits up to 9, which is the least common leading digit (4.6% of the cases). A distribution abides by the Benford’s law if the frequency [*F*(*x*)] of the FSD, *x* ∈ {1, …, 9}, follows the logarithmic relation, $F\left(x\right)=\log_{10}\left(1+\frac{1}{x}\right)$ (1). The law of “anomalous numbers” applies also to the frequency of digits in other positions (1).

Not all the numbers abide by the Benford’s law, but for those which do, violations raise concerns. For example, in accounting and auditing, also at a Governmental level, the Benford’s law has been widely used to detect possible frauds (3–5).

Population-based cancer registries produce a great amount of numbers: the cancer incidence rates. The evaluation of their quality is rather complex, involving different aspects, and it is mainly based on the knowledge of the clinical, diagnostic, and therapeutic pathways of patients and on the process of data collection and registration (6, 7).

The most renowned publication on cancer incidence is Cancer Incidence in 5 Continents (CI5) (8). The cancer registries submitting their data to CI5 have to pass a formal quality evaluation before being accepted. The data quality assessment implies checking the internal coherence, consistency, completeness, and comparability with the final decision taken by a group of experts in the field.

The aim of this study is to evaluate if cancer incidence rates adhere to the Benford’s law to use this mathematical characteristic as a further and objective tool for their quality evaluation.

## Materials and Methods

In the website of the CI5 volume X (CI5-X) (9), the data of the 290 population-based cancer registries included in the publication are available, detailed by all the 424 cancer registry populations (CRPs), as each cancer registry can provide information not only for the whole population but also for different racial and/or ethnic subgroups within the same population.

The CI5-X data include aggregated information for 244 combinations of cancer site and morphological group, specified for 19 age groups (5-year age groups from 0–4 to 85+, plus unknown age) and for the two sexes.

We drew a pseudorandom sample of 10% of the available CRPs, stratified by continent (considering South and North America separately), setting a random number seed to make the sampling reproducible.

Overall, 43 CRPs (from 40 cancer registries) were sampled and included in the analysis: 1 from Africa (Malawi, Blantyre), 3 from Central and South America (Argentina, Tierra del Fuego; Brazil, San Paolo and Ecuador, Quito), 18 from USA (Virginia, Asian and Pacific Islanders; Nebraska, Black; Ohio; Vermont; Montana; Michigan; Georgia; Indiana, White; Missouri, White; NPCR-National program of cancer registries – including 42 States; Colorado, Asian and Pacific Islanders; Arkansas, Black; Alabama, White; Arkansas, White; California, Asian and Pacific Islanders; Connecticut, Black; Virginia, Black; and California), 7 from Asia (India, Karunagappally; Singapore, Malay; Turkey, Edirne; Israel, Jews; Japan, Hiroshima Prefecture; Japan, Fukui Prefecture; and Israel), 11 from Europe (France, Isère; Germany, North Rhine – Westphalia; France, Hérault; UK, England; Estonia; Switzerland, St Gall-Appenzell; Bulgaria; Malta; Ukraine; Spain, Navarra; and Italy, Sondrio), and finally 2 from Oceania (New Zealand; Other and USA, and Hawaii).

The cancer data corresponding to the age group 19 (age unknown) were excluded from the analysis.

After the exclusion of those combinations of cancer morphology and site with no cases, 146,590 combinations were included in the analysis.

Crude incidence rates were computed for each sex, age group, and topography and morphology combination dividing the number of cases by the corresponding population, and expressed per 100,000 inhabitants. The FSD distribution for crude incidence rates was then calculated for all the CRPs together, by sex, and by CRP. Moreover, a sensitivity analysis has been performed randomly excluding half of the most important cancer sites (prostate, lung, breast, and colon–rectum).

For checking the adherence of observed FSD distributions to the Benford’s one, we used different methods.

Since the Benford’s distribution has mean greater than median and is positively skewed (10), these figures have been evaluated for cancer incidence rates.

Theoretical and observed distributions were plotted for a graphical comparison.

According to the literature, we did not use those tests (e.g., χ^2^ and the Kuiper’s statistic) that are extremely sensitive in rejecting the null hypothesis (being a distribution Benford-like) for large samples (4, 11–13). To test the goodness of fit, we used the following tests:

– *r*: the Pearson correlation. This is commonly used to measure how closely a distribution follows the Benford’s law (11, 12). The most the coefficient “*r*” is close to +1 the highest the correlation between Benford’s law and the observed FSD distribution is.
– χ^2^/*n*: the χ^2^ divided by the sample size (4, 14).
– *m*: the maximum distance in absolute terms between expected and observed frequencies for each of the nine digits (1–9). The statistics may vary between 0 (no differences between the two distributions) to +∞ (maximum difference) and the corresponding formula is *m* = max_i = 1, 2, …, 9_{|*b*_i_−*e*_i_|} (12), where *b*_i_ is the frequency expected by Benford and *e*_i_ is the observed frequency for each digit i.
– d*: the normalized Euclidean distance between the two distributions divided by the maximum possible distance, which would occur when the FSD was 9 for all the numbers. The corresponding formula is:
  $$d^{*}=\sqrt{\sum_{\text{i}=1}^{9}{\left(b_{\text{i}}-e_{\text{i}}\right)}^{2}}∕\sqrt{\sum_{\text{i}=1}^{8}{\left(b_{\text{i}}\right)}^{2}+{\left(1-e_{9}\right)}^{2}}$$
  where *b*_i_ is the frequency expected by Benford and *e*_i_ is the observed frequency for each digit i. The statistic may vary between 0 (no differences) to 1 (maximum difference) (12).
– *Z* statistic: the average of the *Z* values for each comparison between the nine observed and theoretical digits distributions (5):
  $$Z=\frac{1}{9}\sum_{\text{i}=1}^{9}\sqrt{n}\left[\frac{|b_{\text{i}}-e_{\text{i}}|-1∕\left(2n\right)}{\sqrt{b_{\text{i}}\left(1-b_{\text{i}}\right)}}\right]$$
  where i = 1, …, 9 is a fixed digit, *b*_i_ is the frequency expected by Benford, and *e*_i_ is the observed frequency for each digit i. The cut-off value for statistical significance, with alpha = 0.05 and one side tail, is 1.64.

For providing an inter-CRP comparison, the mean, the median, and the 10th or the 90th (the one including the most extreme values) percentile of each statistic were computed.

A summation index has been computed for rating the CRPs according to the statistics’ results. Each CRP received one point for each statistic in the 10th or 90th percentile (whichever represents the worst values). The summation index could vary from 0 (no statistic beyond the threshold) up to 5 (all statistics beyond the threshold). The probability for each statistic to be in the most extreme decile was 0.1 (approximately 4/43) assuming independence between statistics, considering that the summation index follows a binomial distribution (pr = 0.1, *n* = 5) the random probability for a CRP to have the summation index equal to 0 is 0.59, to 1 is 0.33, to 2 is 0.07, to 3 is 0.008, to 4 is 0.0005, and to 5 is 0.00001.

The analysis has been performed with Stata v. 12, using specific commands for extracting the sample (“sample” and “seed”) and for computing observed and Benford FSD distributions (“digdis”).

## Results

When considering all the cancer incidence rates together (146,590 observations), the distribution of the FSDs appeared to be positively skewed (0.84), with the mean (3.38) greater than the median (3.0). These values were close to those of the theoretical Benford’s distribution (skewness 0.8, mean 3.44, and median 3.0), as were the ratios between 1st vs. 9th (observed 6.6 vs. Benford 6.6), and between 1st vs. 2nd (1.8 vs. 1.7) FSD.

**Figure 1:**
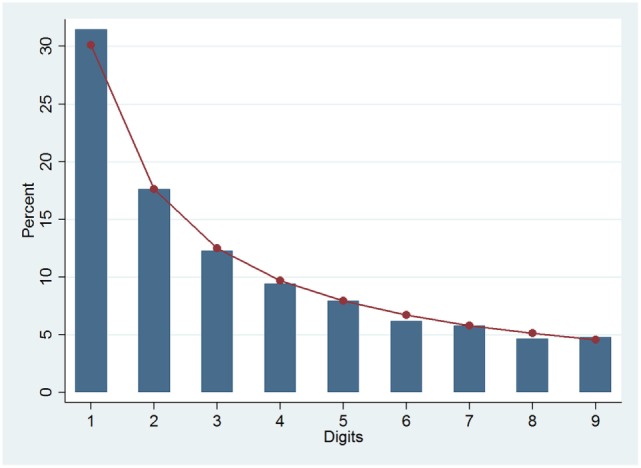
**Benford (line) and observed (columns) distributions of first digits for all crude cancer incidence rates**.

These results let suppose that the FSD distribution of cancer incidence rates might adhere to the Benford’s pattern. In fact, when the observed FSD distribution was graphically compared to the theoretical one, as shown in Figure 1, they were almost overlapping.

The Pearson’s correlation coefficient, *r*, showed an almost perfect direct correlation between the observed FSD distribution and the expected one (0.999); moreover, all the measures of the distance between the distributions were very low (*m* = 0.014 and d* = 0.015), and the average *Z* was below the significance level. Finally, the χ^2^ test, weighted on the number of observations (χ^2^/*n*), was also very low (0.002).

The analysis has been repeated by sex and confirmed the same results (data not shown). Also, after the exclusion of half of the rates for the major cancer sites were excluded the overall results confirmed the adherence of the FSD distribution to Benford’s law (*r* = 0.999, *m* = 0.014, d* = 0.016, χ^2^/*n* = 0.002).

When single CRPs were evaluated, each FSD distribution was positively skewed, and the mean was greater than the median (data not shown).

**Figure 2:**
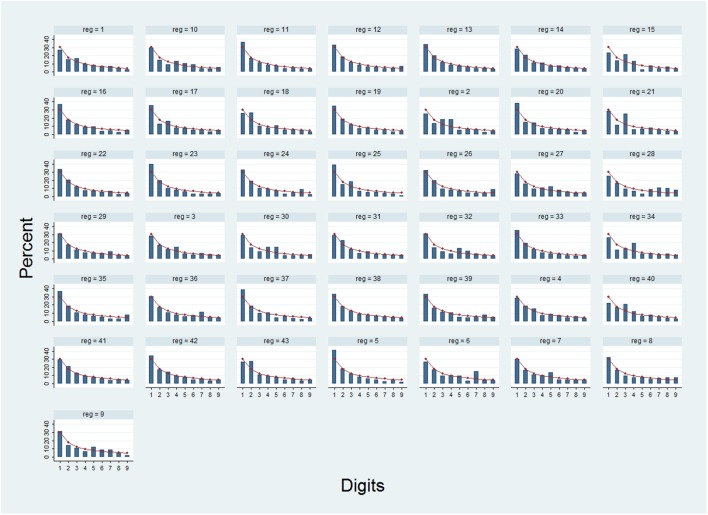
**Theoretical (line) and observed distributions (columns) of first digits for all the analyzed incidence rates, by registries and populations (reg)**. 1: Malawi, Blantyre; 2: Argentina, Tierra del Fuego; 3: Brazil, San Paolo; 4: Ecuador, Quito; 5: USA, Virginia, Asian and Pacific Islanders; 6: USA, Nebraska, Black; 7: USA, Ohio; 8: USA, Vermont; 9: USA, Montana; 10: USA, Michigan; 11: USA, Georgia; 12: USA, Indiana, White; 13: USA, Missouri, White; 14: USA, NPCR-National program of cancer registries (including 42 States); 15: USA, Colorado, Asian and Pacific Islanders; 16: USA, Arkansas, Black; 17: USA, Alabama, White; 18: USA, Arkansas, White; 19: USA, California, Asian and Pacific Islanders; 20: USA, Connecticut, Black; 21: USA, Virginia, Black; 22: USA, California; 23: India, Karunagappally; 24: Singapore, Malay; 25: Turkey, Edirne; 26: Israel, Jews; 27: Japan, Hiroshima Prefecture; 28: Japan, Fukui Prefecture; 29: Israel; 30: France, Isère; 31: Germany, North Rhine – Westphalia; 32: France, Hérault; 33: UK, England; 34: Estonia; 35: Switzerland, St Gall-Appenzell; 36: Bulgaria; 37: Malta; 38: Ukraine; 39: Spain, Navarra; 40: Italy, Sondrio; 41: Germany, Brandemburg; 42: New Zealand: Other; 43: USA, Hawaii.

In Figure 2, the FSD distribution of all cancer incidence rates and the Benford distribution were compared for each of the 43 analyzed CRPs. The shapes of all distributions generally resembled the Benford’s one, with a decreasing percentage of FSD from 1 to the 9. However, a few possible differences were shown.

The Pearson correlation coefficients were very high for the majority of the CRPs (median = 0.97); however, some values were relatively low (0.85 representing the 10th percentile). Also, the other measures of distance were generally low (median: *m* = 0.05, d* = 0.07), but still the corresponding 90th percentiles reached rather higher values (0.10 and 0.12, for *m* and d*, respectively). For the ratio between the χ^2^ and the number of rates, the 90th percentile was almost 3-time the median (90th percentile = 0.14 and 50th percentile = 0.05), and, finally, for the average *Z*, the value of the 90th percentile corresponded to the value of statistical significance (1.64).

Although the majority of the CRPs reported statistics showing an agreement with the Benford’s law, for a few of them, the values seemed to indicate possible discrepancies.

For 35 CRPs, the summation index was 0, for 4 CRPs was 1, and for 2 CRPs was 2. Only one CRP (Argentina, Tierra del Fuego) reported a summation index of 3 (*r* = 0.839; d* = 0.125; χ/*n* = 0.146) and another one (USA, Virginia, Black) had all the five statistics in the worst classes (*r* = 0.82; *m* = 0.13; d* = 0.147; χ/*n* = 0.182; *Z* = 1.88). The probability for the two latter results to happen by chance is very low. Therefore, for such CRPs, a possible violation of the Benford’s law should be considered.

## Discussion

In the present study, a considerable and heterogeneous sample of CRPs, included in CI5-X, was analyzed to evaluate, for the first time to our knowledge, if the FSD distribution of cancer incidence rates abided by the Benford’s law.

The results showed a substantial adherence of FSD distribution of cancer incidence rates to the Benford’s law.

This was not surprising. In fact, FSD distribution of cancer incidence rates had *a priori* some characteristics for being Benford prone. Indeed, they are the second generation distribution, being the result of the division of the number of cases diagnosed in a time span by the corresponding resident population, they comprise a large range of numbers covering several orders of magnitude (from units to thousands per 100,000 people, according to different ages and cancer types), and they are not influenced by human thought (15, 16).

We verified that cancer incidence rates respect the quantitative measures suggested by Wallace (10) to assess whether a distribution may be expected to obey the Benford’s law. In fact, the mean of their observed FSD is greater than the median, and their distribution has a positive skewness.

In the present study using graphical visualization, correlation coefficient, and some distance statistics, we observed that FSD distribution of cancer incidence rates abide by the Benford’s law when analyzed overall, by sexes, excluding half of the rates for the major cancer sites (female breast, colon–rectum, and lung and prostate cancers) and generally by CRP.

We have analyzed data which had been already examined for their quality and proved as good for publication in CI5-X (8). Therefore, no major problems in data quality were expected. In fact, our results showed that for almost all the CRPs the FSD distribution substantially adhere to the Benford’s law. When the 43 CRPs were analyzed individually, the plot of their FSD distribution seemed to be in agreement with the Benford’s law. It must be mentioned that, due to sampling, two CRPs were subgroups of the same registry (USA, Arkansas Black and White; USA Virginia, Asian and Pacific Islanders and Black) and two others included a subgroup and the whole population of the same registry (Israel and Israel, Jews and USA California and USA California, Asian and Pacific Islanders). No large difference within those cancer registries has been shown. Therefore, quality of cancer registry data and related activity (in terms of data availability, data collection, etc.) seemed not related to racial/ethnic subgroups at least in the analyzed registries.

The cancer registry data quality evaluation is not a perfect process, and some residual heterogeneity could exist also in CRPs included in CI5-X. In fact, in the introduction of CI5-X, it is stated that in the registry specific pages for some CRPs “an asterisk preceding the registry title indicates that special considerations (which may include underregistration) must be taken into account in interpreting the published rates or indicators of quality…” (8). Overall, asterisks were reported for 114/424 CRPs in CI5-X (26.9%), and in 11/43 (25.6%) in our sample. One of the two CRPs which had three or more statistics with the worst values for Benford’s compliance had the asterisks (50%), in comparison with the others in the sample (10/31, 24.3%).

We evidenced that, although the majority of CRPs seemed to adhere to the Benford’s law, at least two of them showed possible violation. Random fluctuations could have driven the observed results (14), even if with a very low probability, but the coherence across the different applied statistics made, for these CRPs, the inconsistency with the Benford’s distribution more probable.

According to our experience, based on the analyzed dataset that has been already checked for data quality and accepted for publication (CI5-X), cancer registries showing the poorest results had *r* value below 0.9 and *m*, χ^2^/*n*, and d* values higher than 0.10; presumably in a wild situation, greater values are expected.

The adherence to Benford’s law has been widely used not only to detect fraudulent data in business and administration (3) but also to test data irregularities in scientific research (17). Frauds in cancer incidence data are not expected. However, non-adherence to the law may be a clue for further evaluation. The distance from the expected distribution may be the consequence of selections or incompleteness of the data collection, of rounding of small rates (18), of errors in data recoding or in data transfer.

The meaning of Benford’s violation is a red flag showing an unusual behavior requesting further data examination (14). Once the suspect for a violation is raised, a CRP, which owns more data than those we analyzed, should try to find out clues for the possible problem. Our suggestion is to look for the Benford patter for incident cases based on different (combinations of) sources of information (pathology reports, hospitalization, death certificate, etc.) to detect any source-specific pattern. Moreover, it should be evaluated the stability over time of the data flow, for each information source and cancer site.

## Conclusion

Checking for adherence to the Benford’s law is not suggested in place of the traditional cancer registry data quality process, but it could be used as a simple and objective tool in the first steps to identify those cancer registries to be evaluated with great attention.

## Author Contributions

EC conceived the idea of the study, planned and designed it, and drafted the first draft. GR made substantial contribution to the statistical analysis and revised critically the paper. Both authors edited and approved the final version of the manuscript. Both authors are accountable for all aspects of the work.

## Conflict of Interest Statement

The authors declare that the research was conducted in the absence of any commercial or financial relationships that could be construed as a potential conflict of interest.
